# Pathway of effects of adverse childhood experiences on the poly-drug use pattern among adults using drugs: A structural equation modeling

**DOI:** 10.3389/fpubh.2023.1043222

**Published:** 2023-04-05

**Authors:** Jing Li, Jianhui He, Pei Wang, Jiashuang Li, Yunjia Zhang, Jing You, Virasakdi Chongsuvivatwong

**Affiliations:** ^1^School of Public Health, Kunming Medical University, Kunming, Yunnan, China; ^2^NHC Key Laboratory of Drug Addiction Medicine, Kunming Medical University, Kunming, Yunnan, China; ^3^International Research Fellow, Prince of Songkla University, Hat Yai, Songkhla, Thailand; ^4^School of Public Health, Fudan University, Shanghai, China; ^5^Chenggong Campus, Yunnan University Secondary School, Kunming, Yunnan, China; ^6^Department of Infectious Diseases and Hepatology, The First Affiliated Hospital of Kunming Medical University, Kunming, Yunnan, China; ^7^Epidemiology Unit, Faculty of Medicine, Prince of Songkla University, Hat Yai, Songkhla, Thailand

**Keywords:** poly-drug uses, adverse childhood experiences (ACEs), structural equation models (SEM), drug use, addiction

## Abstract

**Introduction:**

Adverse childhood experiences (ACEs) are associated with an increased risk of poly-substance use among drug-using adults. However, there is a paucity of literature on a direct or indirect relationship between ACEs and drug use patterns. We thus aimed to identify the pathway of effects of ACEs on drug use patterns in adults by the structural equation model (SEM).

**Methods:**

A cross-sectional study was conducted by respondent-driving sampling and consecutive sampling among adult drug users in Southwest China in 2021. Descriptive, univariate, and SEM analyses were performed by R software 4.2.1.

**Results:**

Of 406 participants recruited from a drug abuse clinic, the average age was 34 years. The majority of the participants were male patients (98.3%) from ethnic minorities (79.6%), who were unmarried (71.6%) and employed (81.2%). Nearly 95.5% experienced ACEs with 46.6% of them reporting four or more ACEs. The median value of self-perception of drug abuse score, friend drug use score, and drug use score was 8.0 (3.0, 11.0), 1.0 (0.0, 1.0), and 1.0 (1.0, 2.0) respectively. In the confirmatory analysis part of SEM, the construct of latent variables fitted well with the data. Poly-drug use was significantly and directly affected by three predictors including monthly incomes (β = 0.09), friend drug use (β = 0.50), and ACEs (β = 0.11). The indirect effect of ACEs passing through self-perception of drugs (β = 0.09) was not significant.

**Discussion:**

ACEs have an independent and direct effect on the drug user for poly-drug use apart from the effect of drug-using friends and family income.

## 1. Introduction

Drug use could result in a higher risk of negative health outcomes (1), such as anxiety depression, unprotected sexual behavior, and sexually transmitted diseases (2) with increased mortality (3) among drug users. The estimated number of people aged 15–64 using drugs has increased globally from 226 million to 274 million indicating that drug use was increasing (4). Categories of substances expanded, including cannabis and stimulants, with over 22 million European adults (5), and the prevalence ranged from <5% in Malta and Slovenia (1) to more than 95% in Cyprus and Poland. In China, 40.6%–80.0% of drug users co-used alcohol, depressants, marijuana, stimulants, and even new psychoactive substances (NPS) (6). In addition, the prevalence of 72.7% and 17% for tobacco and alcohol co-usage was higher than a single-use population with 20.8% and 2.2%. Meanwhile, it was 59.8% of poly-drug use compared to 16.7% for mono-pharmacy seen in China (7). Poly-drug use was more neurotoxic than mono-drug use with addictive effects including perception deficits and health problems (8). However, there was a lack of predictors at the early edge of a lifetime to prevent drug use in adults. An additional sample of participants was obtained.

Adverse childhood experiences (ACEs) are associated with an increased risk of substance use, even the development of drug-use disorders (9). Recent evidence showed that the prevalence of ACEs is increasing. It ranged from 85.4% to 100% (10) among drug users compared to 66.2%–75% among non-drug users (11, 12). Poly-drug users frequently used tobacco during their youth, as well as with the increasing prevalence of tobacco and cannabis co-use, and the excessive use of alcohol without other drug use (13). A previous study conducted in China found an association between ACEs and injecting drug-use behaviors with amphetamine use (14). However, the causation between ACEs and adult drug use remained unclear, and the evidence of a direct or indirect association between ACEs and poly-drug use was lacking.

In addition to ACEs, potential risk factors were possibly associated with drug use behaviors including socio-economic characteristics, alcohol (15) and tobacco abuse (16), self-perception of drug use, family environment and parental rearing styles (17), and friends' drug use (15). Association with health status among poly-drug users was found to have extensive socio-economic marginalization as well as sexual risk behaviors in Brazil (18), and family or friend drug use perhaps led to more frequent or heavier use of each substance (19). Meanwhile, isolation or a shortage of social support possibly tended to be experienced by poly-drug users (20). Although the evidence showed complicated relationships among them, it is unclear about both direct and indirect effects as well as causal paths among these possible risk factors on drug use, especially ACEs effects on poly-drug use in adulthood. As ACEs, probably being a predisposing predictor in early life for drug use in adulthood, it was necessary to explore the pathway of ACEs effects on adult substance use with other controlled factors affecting multiple substance use.

**Figure 1:**
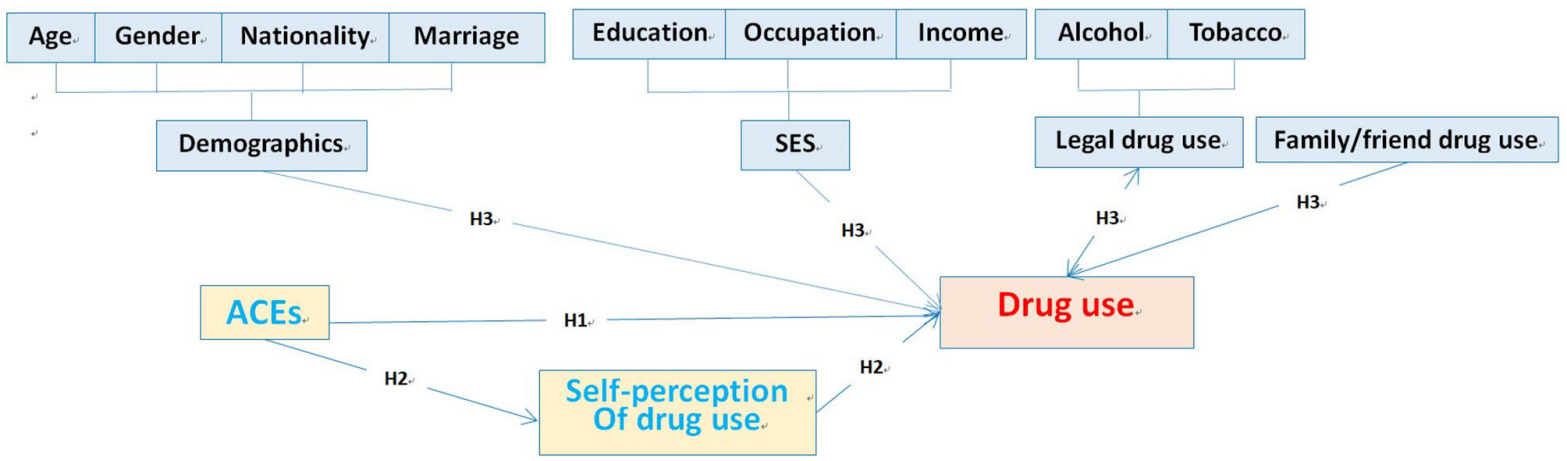
Hypotheses of the study.

This study intended to determine whether and how exposure to adverse experiences during early childhood directly or indirectly predicted the poly-drug use pattern among drug-using adults. We argued that adverse childhood experiences may affect the pattern of drug use by the intermediary variable. Structural equation modeling (SEM), a more advanced form of regression analysis, was an important analytical strategy to disclose the effects of a variable, such as experiences in an early lifetime on the drug use pattern (21). It was used to enhance our understanding of the pathway from ACEs to the final drug-use behavioral outcome in adulthood. If this intermediary variable is confirmed, and the reasons for drug-use patterns can be well documented, future interventions should emphasize experiences in the early lifetime to improve and prevent drug use, especially poly-drug use during adulthood. Reported findings led to the development of the current hypothesis: (1) exposure to ACEs could predict the subsequent development of drug use even poly-drug use in adulthood; (2) identify the pathway of the influence of ACEs on adult drug-use pattern, and (3) socio-economic characteristics would modify the effects on the development of drug use in adulthood by ACEs (Figure 1).

Our general objective was to constitute an extension of previous studies to increase the understanding of the relationship between drug consumption and prior childhood maltreatment. Specifically, we explored the pathways of the influence of ACEs on the drug-use pattern in adulthood and the possible moderating effects of socio-economic characteristics in samples from Southwest China.

## 2. Methods

### 2.1. Study setting

Our study site was a drug-treatment center in the Jinghong city of Xishuangbanna Autonomous Prefecture, Yunnan Province, where the problem of drug abuse was most prevalent in the province from January to July 2021. Participants were selected by consecutive sampling among drug-using adults who voluntarily came for service. All the participants, the majority of whom local to the province, could communicate in Chinese. An additional sample of participants was obtained from Response Driven Sampling in order to cover participants who were not attending the service. Inclusion criteria were those who had injection drug use in the lifetime and/or used stimulants in the past 6 months, and who were more than 18 years old. Those who could not participate due to physical and psychological reasons were excluded. The 406 eligible participants mainly used self-made questionnaires. The details of the study setting, participants, sampling, and data collection processing can be seen in our previous study (22).

### 2.2. Measurements

Questionnaires were used to measure sociodemographic data, personal drug use history, family and peer substance use histories, self-perception of drug use, and categories of illegal drug use. The information on the duration of use (in months), frequency of use, dose per use, and mode of administration were also collected. Substance use in our study met the diagnostic criteria for substance dependence in the Psychiatric Diagnostic and Statistical Manual (Fifth Edition) (DSM-V), in which participants were instructed to respond to each question whether they used illegal drugs including opium, heroin, morphine, cannabis, ketamine, crystal meth, meth tab, and ecstasy by giving “no” or “yes” and assigning values from 0 to 1. In addition, family and peer factors included legal (alcohol and tobacco) and illegal substance use of the family members and their friends were also measured.

The self-perception of drug use questionnaire included 20 items covering contents about stigma perception directly from drug use and psychiatric symptoms caused by substance use based on literature (23). ACE questions were developed by using a standardized protocol from the childhood trauma questionnaire (CTQ-SF) (24)and part of items from the National Survey on Alcohol and Related Conditions (NESARC-III, USA) was initially treated as an intermediary variable (25). We first selected nine items of questions related to the experience of adverse events from the family. These included neglected daily life care, language insults, events of sexual abuse, failure to provide medical care, emotional neglect, existence dis-identification, physical violence, unconcerned family atmosphere, and family disorders due to substance use.

These questions were initially translated into Chinese and modified by the main researcher to suit the Chinese context. A team of experts including two chief physicians from departments of neurology and infectious disease separately and three experts in drug dependence prevention at the Institute of Drug Dependence of Yunnan Province reviewed and finalized the Chinese questions. The finalized version was back-translated into English and compared with the original version to establish the validity of the Chinese version. In-depth interviews were conducted with five border drug users to obtain cultural and contextual relevance. The respondents were asked specific related questions to determine whether the nine questions were understandable and whether the intent of each question was accurately conveyed. They were also asked to elaborate on the reasons why a positive response category was chosen. Based on their suggestions, we modified the questionnaire for clearer comprehensibility and cultural suitability. In January 2021, a pilot study was conducted among 20 border drug users of methadone clinics and detention centers. The participants responded to each question of the questionnaire (ACEs, self-perception, and family/peer factors) by giving “no” or “yes” and similarly assigning values from 0 to 1.

### 2.3. Variables

The dependent variable was drug use scores ranging from 0 to 8. Socio-demographic variables included age, gender, ethnicity, and marital status. Indicators of socio-economic status (SES) included occupation, education, and monthly income. Among them, age and average income were continuous variables. Ethnic groups were classified into two categories: Han and ethnicity. Marital status was grouped into two categories: (i) unmarried (cohabiting, divorced, and widowed) and (ii) married. For SES factors, education was grouped into two levels: (i) primary school or less and (ii) high school and above. The occupation was grouped into two levels: (i) unemployed and (ii) employed (mainly engaged in agriculture or workers).

In addition, ACE scores ranging from 0 to 9 were used to evaluate the cumulative levels of multiple ACEs with a higher score indicating more serious exposure to ACEs. It can be divided into four levels (no exposure = 0, mild = 1–2, moderate = 3; and severe= equal to and more than 4). The friend drug and family member drug use scores were both from 0 to 8, and the scores of self-perception of drug use ranged from 0 to 20.

### 2.4. Data management and statistical analysis

Data management was carried out using Epidata 3.1, and data analyses were conducted using R software 4.2.1. The chi-square test and rank sum test were used for the comparison of risk levels in the univariate analysis. Structural equation model (SEM) analysis was used to explore associations and direct/indirect effects among variables. A *P*-value of <0.05 was considered to be statistically significant. Finally, the goodness of fit test was used to examine whether the model fits well with the data.

## 3. Results

### 3.1. Distribution of socio-economic-demographics

399 of the 406 drug users were male, with an average age of 33 years, 79.5% of ethnic minority, 91.6% of drinking, 71.6% of unmarried and employed accounting for 81.2%. A total of 95.5% of respondents experienced ACEs with 46.4% reporting four or more ACEs. The median values (inter-quartile range) for monthly income, self-perception of drug abuse score, friend drug use score, and drug use score were 3500.0 (2500.0, 5000.0), 8.0 (3.0, 11.0), 1.0 (0.0, 1.0), and 1.0 (1.0, 2.0), respectively (Table 1).

**Table 1:** Socio-economic-demographics and respondents' variables.

**Variables**		***N* = 406**	**%**
Age (Mean±sd.)		34.0 ± 10.0	
Gender	Male	399	98.3
	Female	7	1.7
Nationality	Han	83	20.4
	Minority	323	79.5
Marital status	Unmarried	291	71.6
	Married	115	28.4
Education	≤Primary school	207	50.9
	≥Junior school	199	49.1
Occupation	Unemployed	76	18.8
	Employed	330	81.2
Income [Median(P_25_,P_75_)]		3500.0 (2,500.0, 5,000.0)	
Category of ACEs	0	18	4.4
	1–2	105	25.9
	3	94	23.2
	≥4	189	46.4
Drinking	No	34	8.4
	Yes	372	91.6
Smoking	No	8	2.0
	Yes	398	98.0
Friend drug use score [Median(P_25_,P_75_)]		1.0 (0.0, 1.0)	
Self-perception of drug abuse score [Median(P_25_,P_75_)]		8.0 (3.0, 11.0)	
Drug use score [Median(P_25_,P_75_)]		1.0 (1.0, 2.0)	

### 3.2. Correlations between variables in SEM

Age was negatively related to income, drinking status, and ACEs (*p* < 0.01); friends' drug use was positively associated with the drug use summary score (*p* < 0.001); the score was negatively correlated with ACEs (*p* < 0.05); self-perception of drug abuse was positively related to ACEs (*p* < 0.001), and ACEs were positively related to drinking status (*p* < 0.05) (for details, refer to Table 2).

**Table 2:** Correlations between variables in the SEM model.

	**Age**	**Income**	**Friend**	**Family**	**Smoking**	**Drinking**	**Self-perception**	**ACEs**	**Drug-use**
Age	1.000	−0.203^**^	0.081	0.019	−0.085	−0.156^**^	−0.039	−0.129^**^	−0.014
	–	0.000	0.103	0.700	0.088	0.002	0.431	0.009	0.771
Income	−0.203^**^	1.000	−0.027	−0.056	−0.022	−0.039	−0.042	0.025	0.078
	0.000	–	0.589	0.258	0.654	0.431	0.396	0.612	0.118
Friend	0.081	−0.027	1.000	0.152^**^	0.112^*^	−0.002	0.086	0.043	0.381^**^
	0.103	0.589	–	0.002	0.024	0.965	0.083	0.384	0.000
Family	0.019	−0.056	0.152^**^	1.000	0.043	−0.035	−0.104^*^	−0.035	0.125^*^
	0.700	0.258	0.002	–	0.389	0.477	0.036	0.481	0.012
Smoking	−0.085	−0.022	0.112 ^*^	0.043	1.000	0.213^**^	0.113^*^	0.140^**^	0.159^**^
	0.088	0.654	0.024	0.389	–	0.000	0.022	0.005	0.001
Drinking	−0.156^**^	−0.039	−0.002	−0.035	0.213^**^	1.000	0.080	0.098^*^	0.068
	0.002	0.431	0.965	0.477	0.000	–	0.107	0.049	0.170
Self-perception	−0.039	−0.042	0.086	−0.104^*^	0.113^*^	0.080	1.000	0.410^**^	0.025
	0.431	0.396	0.083	0.036	0.022	0.107	–	0.000	0.609
ACEs	−0.129^**^	0.025	0.043	−0.035	0.140^**^	0.098^*^	0.410^**^	1.000	0.116^*^
	0.009	0.612	0.384	0.481	0.005	0.049	0.000	–	0.020
Drug-use	−0.014	0.078	0.381^**^	0.125^*^	0.159^**^	0.068	0.025	0.116^*^	1.000
	0.771	0.118	0.000	0.012	0.001	0.170	0.609	0.020	–

1. ^*^p < 0.05, ^**^p < 0.01, ^***^p < 0.001.

### 3.3. Predictors of poly-drug use by SEM

**Figure 2:**
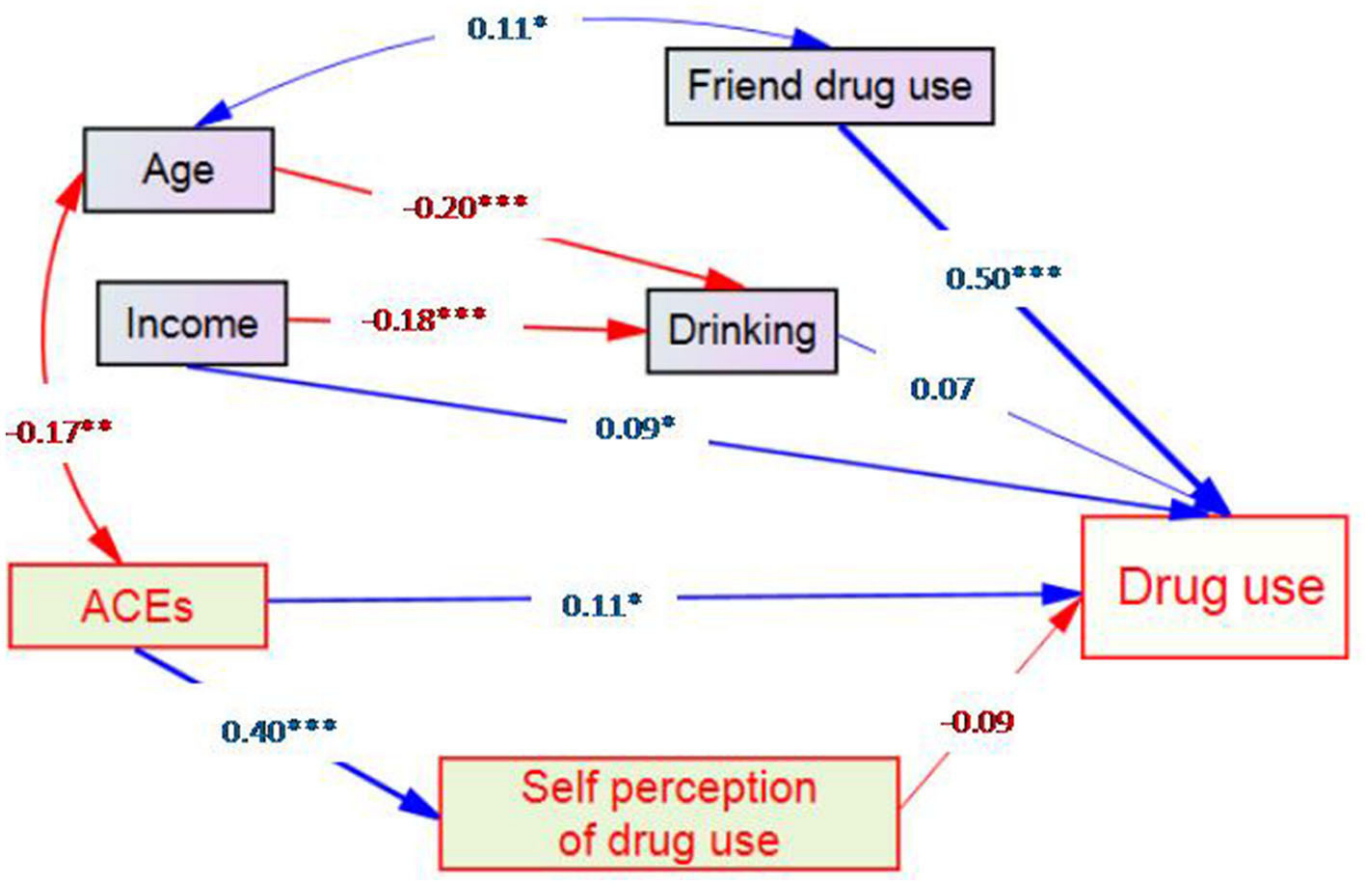
SEM results showing the pathway of effects of adverse childhood experiences on the poly-drug use pattern among drug-using adults (Note: 1). *p < 0.05, ^**^p < 0.01 and ^***^p < 0.001).

From the goodness of fit tests, the final structural model fitted the current data well [chi-square = 10.146 (df = 11, *p* = 0.517), RMSEA = 0.000 (95% CI: 0.000, 0.049), AGFI = 0.979, and CFI = 1.000]. Figure 2 shows that poly-drug use was significantly associated with monthly incomes, friend drug use status, and ACEs presenting a standardized estimate of the model. Poly-drug use was directly affected by three predictors including peer drug use (β = 0.50), ACEs (β = 0.11), and income (β = 0.09). Although ACE had a significant effect on self-perception of drug use (β = 0.40), the effect of the latter on poly-drug use was not significant. Thus, there was no evidence of intermediary effects of self-perception of drug use.

It should be noted that self-perception of drug use was initially treated as an intermediary variable between ACEs and drug use as indicated by one arrow pointing from ACEs to this variable and then from this variable to drug use. However, the coefficient of the latter arrow of −0.09 was not significant. We, therefore, had no evidence that it was an intermediary variable.

## 4. Discussion

In this study, exposure to ACEs could predict the drug-use pattern in adulthood with a direct association. In addition, age, income, and friend drug use also have independent effects on the development of drug use in adulthood.

ACEs, directly contributed to a risky effect on poly-drug uses, and it is consistent with some studies (26). It also demonstrated that exposure to ACEs was significantly associated with drug addiction and poly-drug use (26) with consistent cumulative effects accompanied by categories of substance including short and long effects on initiated drug use and the development of drug use. However, those who have suffered from severe ACEs might not address negative consequences until adulthood, possibly choosing to use drugs to reduce the stress or trauma (26, 27). In other words, as exposure to ACEs increased, the possibility of non-addiction decreased significantly, which may explain why some people use drugs to alleviate the negative effects of childhood trauma to some extent. These possibly were another form of representation of long-term evidence of ACE's prolonging effects.

On the contrary, ACEs were negatively related to self-perception of drug use, but the latter did not significantly link to drug use. An interesting phenomenon found was that a substantive subset of individuals who suffer from ACEs avoid partly or entirely the negative health and social outcomes associated with chronically stressful childhood, a characteristic referred to as resilience (28). It may be that good self-perception of drug abuse fosters negative outcome expectancies associated with drug use and hence reduced the likelihood that poly-drug use was initiated later in life. Self-perception of drug use might result in lower self-efficacy, decreased motivation, and reduced health-related quality of life; and it has been associated with increased depression risk (20). In addition, self-perception could impact substance use outcomes among those at risk of and living with substance use disorder through mediating mechanisms, which possibly led to substance use initiation, regular use, and problem or risky use (29). Furthermore, emerging intelligence suggested a range of factors that can help individuals develop resilience during childhood. Strong links with cultural traditions had better-developed self-regulation skills, and a sense of having some control over personal circumstances had wholly been associated with moderating the negative impacts of childhood adversity (30, 31). Thus, the more ACEs the target population experienced, the more the self-perception of substance abuse they had, and those who had adverse experiences in childhood perhaps nurtured resilience so that ACEs were probably negatively associated with self-perception of substance use.

Income was a positive factor associated with drug use, and it is similar to other studies. Earlier research has found higher-intensity substance use to be associated with greater sex work income (32). Obviously, it is an important determinant of health among drug users commonly with unemployment, who are typically characterized as incapable of and adverse to participation in formal employment (33). There was a positive association between heavy alcohol use and male employment, and a positive association between female counterparts and daily cannabis use (34), based on Kaitlyn Jaffe's models. Our participants with poly-drug use may require access to greater remuneration from income-generating activities.

Friend drug use contributed to a high score of drug use, but family drug use was not associated with poly-drug use. Prior findings about family influences were contradictory to our results (35). Family factors did not have as much influence on our target adult drug users as teenagers. Probably, teenagers were not mentally sound enough and were vulnerable to the impact of the surrounding environment, especially their peers. Moreover, only 8.4% of drug users had family members of drug use in our study, so family influences possibly could not be shown due to the limited sample size. On the other hand, it was consistent for peer influences with our result. Studies suggested that all categories of substance use among adolescents possibly were influenced by peers, (36) especially best friends (37). Peers have common interests and hobbies. They learn from each other and imitate each other's behavior. Especially under peer pressure, some people could not withstand the pressure and tend to take drugs (38). Conclusively, friend drug use accelerated the progressions of drug use to their abuse or dependence.

Our study did not identify the association between tobacco and drug abuse, and alcohol usage was not significantly associated with drug use by the SEM model. It is totally contradictory to other studies, which showed that tobacco (16) and alcohol use (15) were related to drug-abuse behavior, and individuals with addictive behaviors often use multiple addictive substances at the same time, and the use of such multi-addictive substances was not causal (17). However, the univariate analysis of our research showed that smoking was positively related to multi-drug abuse. Possibly, some factors in the model were related to both smoking and drug abuse, which masked the relationship between smoking and drug abuse. Self-perception of drug use was not significantly related to poly-drug use in our study. It is inconsistent with prior results (39). A positive attitude toward drug use was an important prerequisite of usage (40). The more knowledge we gain about the dangers of drug use, the deeper the belief, and the more inclined we become to stay away from drugs.

There were limitations that should be noted. First, the measurement of ACEs relied on self-recall resulting in information bias. Second, the derivation of causality may be restricted due to the cross-sectional design. In addition, pathways suggested by structural equation modeling are not inclusive and barely address directly observed variables without latent variables that may affect the exposure as well as the outcome, which may limit causality explanation. Finally, the study was confined to drug users at the treatment center without a control group who were non-drug users, and the majority of participants were mostly male patients of Dai ethnicity, which limited the generalizability of this study.

## 5. Conclusion

ACEs could be a predictor for the drug-use pattern in adulthood, as well as friends' drug use and income, in which self-perception of drug use as a mediator between ACEs and poly-drug use could not be confirmed. Strategies on how to build a good family environment, minimize childhood stressful and/or traumatic experiences, and improve teenagers' knowledge of drugs and anti-drug skills would be effective to avoid becoming a drug addict. Meanwhile, a cohort study should be carried out for the further exploration of the causality between ACEs and drug use in adulthood.

## Data availability statement

The raw data supporting the conclusions of this article will be made available by the authors, without undue reservation.

## Ethics statement

The studies involving human participants were reviewed and approved by Institutional Review Board of Kunming Medical University. The patients/participants provided their written informed consent to participate in this study.

## Author contributions

JinL and VC designed and developed the study. JH, JinL, PW, and JiaL performed literature reviews, wrote the manuscript, and analyzed and interpreted the data. All authors read and approved the final manuscript.
